# Tumor-infiltrating lymphocytes in melanoma: from prognostic assessment to therapeutic applications

**DOI:** 10.3389/fimmu.2024.1497522

**Published:** 2024-12-06

**Authors:** Meshack Bida, Thabiso Victor Miya, Rodney Hull, Zodwa Dlamini

**Affiliations:** ^1^ Division of Anatomical Pathology, National Health Laboratory Service, University of Pretoria, Hatfield, South Africa; ^2^ SAMRC Precision Oncology Research Unit (PORU), DSI/NRF SARChI Chair in Precision Oncology and Cancer Prevention (POCP), Pan African Cancer Research Institute (PACRI), University of Pretoria, Hatfield, South Africa

**Keywords:** melanoma, tumor-infiltrating lymphocytes, prognosis, adoptive cell therapy (ACT), immunotherapy, immune checkpoint inhibitors (ICIs), CD8+, CD4+

## Abstract

Malignant melanoma, the most aggressive form of skin cancer, is characterized by unpredictable growth patterns, and its mortality rate has remained alarmingly high over recent decades, despite various treatment approaches. One promising strategy for improving outcomes in melanoma patients lies in the early use of biomarkers to predict prognosis. Biomarkers offer a way to gauge patient outlook early in the disease course, facilitating timely, targeted intervention. In recent years, considerable attention has been given to the immune response’s role in melanoma, given the tumor’s high immunogenicity and potential responsiveness to immunologic treatments. Researchers are focusing on identifying predictive biomarkers by examining both cancer cell biology and immune interactions within the tumor microenvironment (TME). This approach has shed light on tumor-infiltrating lymphocytes (TILs), a type of immune cell found within the tumor. TILs have emerged as a promising area of study for their potential to serve as both a prognostic indicator and therapeutic target in melanoma. The presence of TILs in melanoma tissue can often signal a positive immune response to the cancer, with numerous studies suggesting that TILs may improve patient prognosis. This review delves into the prognostic value of TILs in melanoma, assessing how these immune cells influence patient outcomes. It explores the mechanisms through which TILs interact with melanoma cells and the potential clinical applications of leveraging TILs in treatment strategies. While TILs present a hopeful avenue for prognostication and treatment, there are still challenges. These include understanding the full extent of TIL dynamics within the TME and overcoming limitations in TIL-based therapies. Advancements in TIL characterization methods are also critical to refining TIL-based approaches. By addressing these hurdles, TIL-focused research may pave the way for improved diagnostic and therapeutic options, ultimately offering better outcomes for melanoma patients.

## 1 Introduction

**Figure 1:**
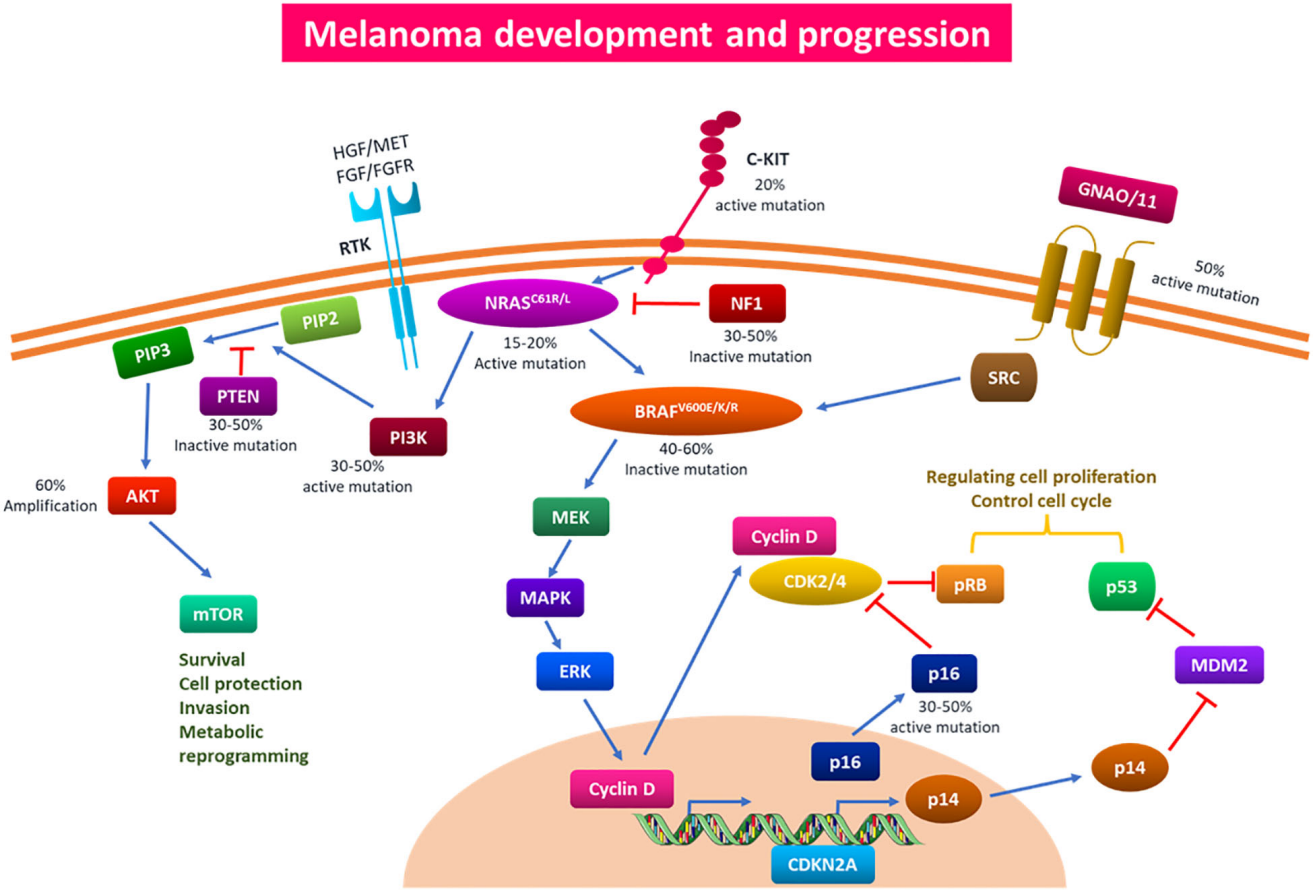
Signaling pathways and genetic mutations implicated in the onset and spread of melanoma. Multiple driver gene alterations are responsible for the genesis and progression of melanoma. Based on particular somatic mutations in various oncogenes, cutaneous melanoma has been classified into four primary subtypes: c-KIT4, GNAQ/11, BRAF, NRAS, NF1, and NF1. Melanoma develops and progresses as a result of these somatic mutations activating the PI3K/AKT and MAPK pathways. Mutations in BRAF can affect the MAPK pathway, which can therefore affect cell cycle regulation and proliferation. The PI3K/AKT and MAPK pathways, which control cell survival, proliferation, invasion, and metabolic programming, can be impacted by mutations in the NRAS gene. In addition to producing medication resistance to traditional therapy, overactivation of the aforementioned pathways can aid in the carcinogenesis, proliferation, invasion, and infiltration of melanoma cells. PI3K/AKT, phosphoinositide 3-kinase/protein kinase B; MAPK, mitogen-activated protein kinase.

Melanoma, the most deadly type of skin cancer, presents a serious clinical issue due to its rapid spread and resistance to traditional treatments. Melanocytes are responsible for this increasingly common condition (1). The tumor, node, and metastasis (TNM) method is used to histologically classify melanoma. Tumors are categorized based on distinct features such as ulceration, lesion thickness, and mitosis. Additional characteristics used to classify melanoma include lymph node involvement and metastasis distance from the source tumor (2, 3). Multiple studies have demonstrated that melanoma has a complicated, multistage genesis that is impacted by hereditary and environmental factors. Numerous benign lesions have been shown to have alterations in the v-raf murine sarcoma viral oncogene homolog B (BRAF) at codon V600E. However, changes in these genes must also occur in other genes participating in various cellular processes for the disease to proceed (4, 5). Target genes, including phosphatase and tensin homolog (PTEN), neurofibromin 1 (NF1), cyclin-dependent kinase inhibitor 2A (CDKN2A), telomerase reverse transcriptase (TERT), and KIT protooncogene receptor tyrosine kinase (KIT), can be mutated in a way that causes benign nevi to become dormant for a long time before developing carcinogenesis. These mutations result in abnormal activation of the PI3K and MAPK pathways, which are physiologically connected to the proliferation and survival of cells (**Figure 1**) (6).

The propensity of melanoma to evade treatment and expand to other organs poses ongoing issues in therapeutics, despite constant advancements in the field (7–9). Arnold et al. (10) predicted that by 2040, there will be a 50% increase in newly diagnosed cases of melanoma. This emphasizes how urgently new treatments are needed to buck this trend (10). Notably, the stage of melanoma at diagnosis largely dictates the treatment approach (**Table 1**). When melanoma is detected in its early stages, the prognosis is very good. Less than 10% of patients with metastases survived for five years, indicating a much higher risk of death. This implies that the primary cause of death linked to melanoma is metastatic illnesses (11).

**Table 1:** A summary table of the 2023 American Society of Clinical Oncology’s guidelines for melanoma therapy standard of care.

Resection status	Melanoma stage	*BRAF* status	Type of Therapy	Neoadjuvant treatment	Primary or Adjuvant treatment	Progression status
Yes	I-IIA	N/A	N/A	Not advised	Not advised	N/A
	IIB/C		Immuno	Not advised	1) nivolumab 2) pembrolizumab	
	IIIA/B/C/D	W-T	Immuno	pembrolizumab for IIIB/C/D	1) nivolumab 2) pembrolizumab	
		Mutant (V600)	Immuno		1) nivolumab 2) pembrolizumab	
			Targeted		Dabrafenib + trametinib	
	IV	N/A	Immuno	pembrolizumab	pembrolizumab	
No	III/IV	W-T	Immuno	N/A	1) ipilimumab + nivolumab, follow with nivolumab 2) nivolumab + relatlimab 3) nivolumab 4) pembrolizumab	Progression on PD-1- based therapies? Use ipilimumab/ipilimumab- containing
		Mutant (V600)	Targeted		1) trametinib + dabrafenib 2) binimetinib + encorafenib 3) cobimetinib + vemurafenib	Progression on BRAF/MEK therapies? Use ipilimumab/ipilimumab- containing
			Immuno		1) ipilimumab + nivolumab, follow with nivolumab 2) relatlimab + nivolumab 3) nivolumab	Progression on PD-1- based therapies? Use BRAF/MEK targeting

N/A, Not available; W-T, Wild type.

Melanoma incidence increased by 320% between 1975 and 2018, with risk variables like nevi count, indoor tanning, UV exposure, and family history all playing a role (12). Although making up a modest portion of cases, melanoma is accountable for roughly 90% of skin cancer deaths. This disease affects both younger and older populations, but elderly people are more likely to experience a rise in incidence. Metastatic spread of melanoma, which initially affects lymph nodes and then most frequently the lungs, is the main source of death from the disease (13). Complete surgical excision is a successful treatment for early-stage melanoma (I–II), with a 99.4% 5-year survival rate (12). However, when melanoma advances, the prognosis gets substantially worse, with stage III and stage IV 5-year survival rates dropping to 68% and 29.8%, respectively (13). Melanoma’s global incidence has been rising, with some countries seeing an annual increase of about 3%. Concomitantly, the disease’s clinical impact is also increasing. According to projections, there would have been 7990 deaths and 97,620 new cases in the United States (US) alone in 2023. The International Agency for Research on Cancer has predicted that in 2020, there would have been 324,635 new instances of melanoma worldwide, along with 57,043 deaths from the illness (14). These figures demonstrate how crucial it is to choose melanoma treatments logically and by the best available data (15).

**Figure 2:**
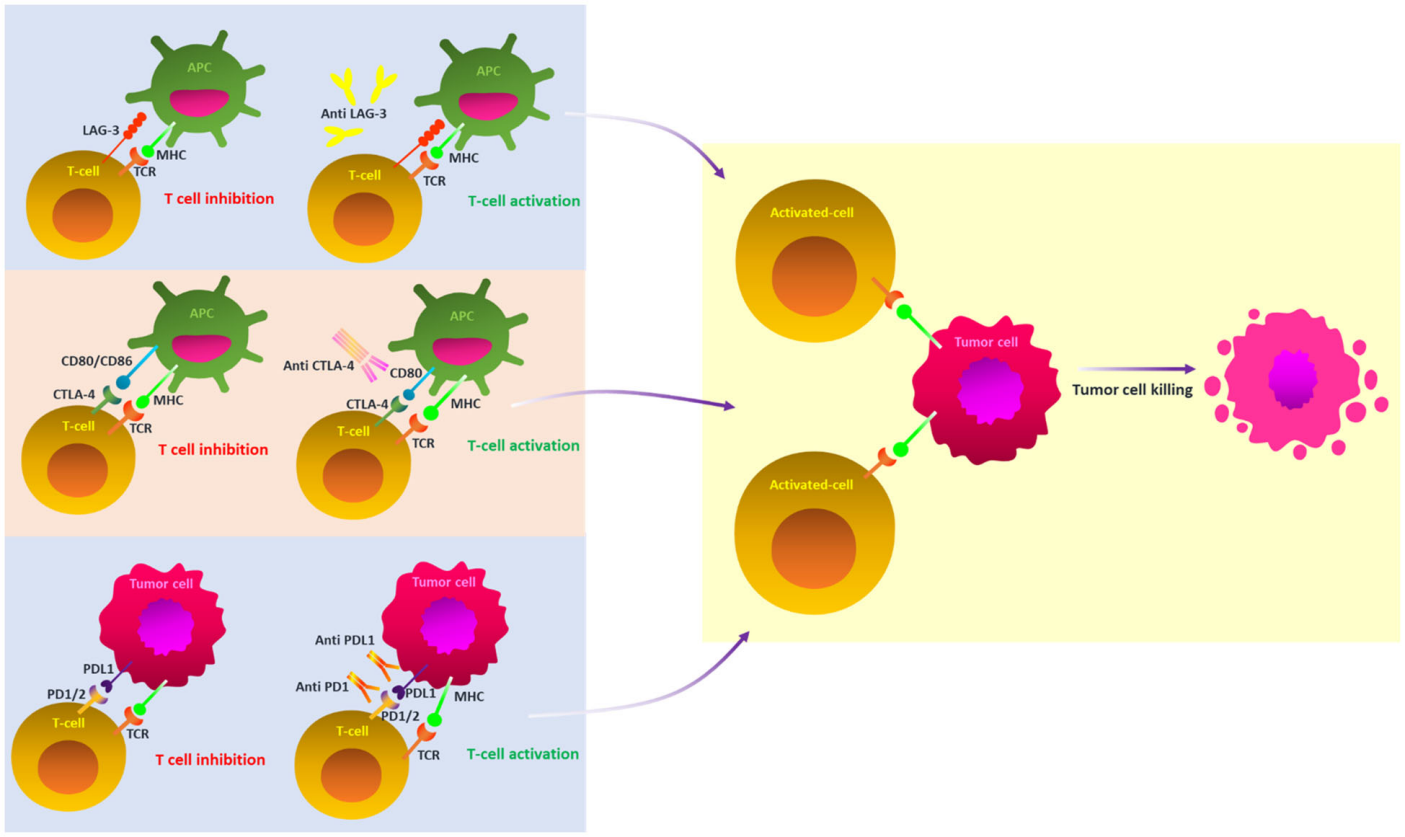
A schematic illustration of key ICI blockade mechanisms in melanoma. Under normal circumstances, the TCR and MHC signaling pathways provide T cell activation; however, these pathways are inhibited by the cooperative action of ICIs inside the tumor microenvironment. ICIs prevent the off signals from being sent by blocking the connections between checkpoints and their companion proteins. This allows the T cells to attack cancer cells. ICI, immune checkpoint inhibitors; PD-1, programmed cell death protein 1; PDL2, PD-2 ligand; PDL-1, PD-1 ligand; CTLA-4, T-lymphocyte antigen 4; TCR, T-cell receptor; MHC, major histocompatibility complex; LAG-3, Lymphocyte-activation gene 3.

Understanding melanoma’s aggressiveness and high death rate requires an understanding of its phenotypic subtypes and gene signatures, which have been greatly enhanced by the progression of genomic technologies, particularly single-cell sequencing (16). The prognosis for metastatic melanoma is still bleak. Even though localized melanoma has a high survival rate, this underscores the significance of efficacious therapy (12). Advances in immunotherapy have completely transformed the way that medication therapy is thought of, creating opportunities for more individualized and efficient care (17, 18). Thus far, immunotherapy has resulted in the creation of immune checkpoint inhibitors (ICIs) (**Table 2**), TME modulators, and oncolytic viral therapy (19, 20). Immune checkpoints are crucial molecules in the cell regulation of the immune response. However, they can be used by tumor cells to subvert immune surveillance (**Figure 2**) (21). Nevertheless, the use of ICIs in particular has drastically increased the long-term survival of melanoma patients in clinical settings and radically changed how they are managed (22). Disappointingly, individuals receiving ICIs frequently only experience temporary advantages, and it can also have harmful side effects (22, 23).

**Table 2:** Key ICIs for treating metastatic melanoma.

Target	Inhibitor	Class
CTLA-4	Tremelimumab CP-675,206	Selective human IgG2 monoclonal antibody
CTLA-4	Ipilimumab (MDX-010)	Selective human IgG1 monoclonal antibody
PD-1/LAG- 3	RO7247669	Bispecific antibody
PD-1	AMP-224	PDL-2 fusion protein
PD-1	AMP-514	PDL-2 fusion protein
PD-1	Pidilizumab (CT-011)	Selective humanized IgG1 monoclonal antibody
PDL-1	Durvalumab (MEDI4736)	Selective humanized IgG1 monoclonal antibody
PDL-1	Avelumab (MSB0010718C)	Selective humanized IgG1 monoclonal antibody
PDL-1	Atezolizumab (MPDL3280A)	Selective humanized IgG1 monoclonal antibody
PD-1	Nivolumab (BMS-936558, MDX- 1106)	Selective human IgG4 monoclonal antibody
PD-1	Pembrolizumab (MK-3475)	Selective humanized IgG4 monoclonal antibody
LAG-3	Relatlimab (BMS-986016)	Selective human IgG4 monoclonal antibody
PDL-1	BMS-936559 (MDX-1105)	Selective human IgG4 monoclonal antibody
LAG-3	Fianlimab (REGN3767)	Selective human IgG4 monoclonal antibody

The immune system is a complex network of cells, organs, and soluble substances that act as the body’s defense mechanism. One cancer treatment that utilizes this capability is immunotherapy, which develops plans to thwart the spread of cancer (24–26). Numerous molecular drugs that can “teach” the immune system to recognize and eradicate tumor cells have been developed as a result of research on immune control’s perspective on tumor evasion mechanisms (27, 28). In this perspective, because of the significant occurrence of lymphocytic infiltrates, metastatic melanoma is regarded as an ideal instance of immunogenic malignancies (17). Metastatic melanoma patients had an average life expectancy of roughly nine months before the introduction of immunotherapy in 2011. These days, 20% of melanoma patients survive for 10 years after being diagnosed. This is due to the discovery of novel treatment targets and the creation of novel immunotherapy medications (29). ICIs (30–32); adoptive cell therapy (ACT) (33); biological drugs, including interferons, stimulating factors, cytokines (34); and vaccines (34) are among the immunological treatments for melanoma.

ACT encourages the direct isolation of immune cells from patients for therapeutic purposes. These cells can be easily multiplied or genetically altered to improve their ability to combat cancer after being isolated. ACTs are always changing and encompass a variety of techniques: 1) TIL therapy; 2) engineered T cell receptor (TCR) therapy; and 3) chimeric antigen receptor (CAR) T cell therapy. The first cellular treatment for a solid malignancy, lifileucel, was approved by the US Food and Drug Administration (FDA) on February 15, 2024. The patient’s own TILs are used in this ACT immunotherapy. These TILs are isolated from a growing tumor that has been removed, grown to a large number in the lab using the T cell growth factor interleukin-2 (IL-2), and then reinfused into the same patient under specific conditions to target tumor cells. As they circulate within the cancer-bearing patient, these TILs can multiply thousands of times, making them a “living drug.” After a multicenter single-arm study in 73 patients (NCT02360579) demonstrated a cancer regression rate of 31.5% by established oncologic criteria, approval was given for adult patients with advanced melanoma that is unresponsive to other successful treatments (35, 36).

TIL therapy has shown promise in treating advanced melanoma in patients who previously received treatment from other therapeutic methods (BRAF/MEK inhibitors or ICI treatments) (37). As a result, this review explores the prognostic significance of TILs in melanoma. It also examines the processes by which TIL influences melanoma outcomes, clinical consequences, and existing challenges and limitations.

## 2 Tumor-infiltrating lymphocytes: an overview

**Figure 3:**
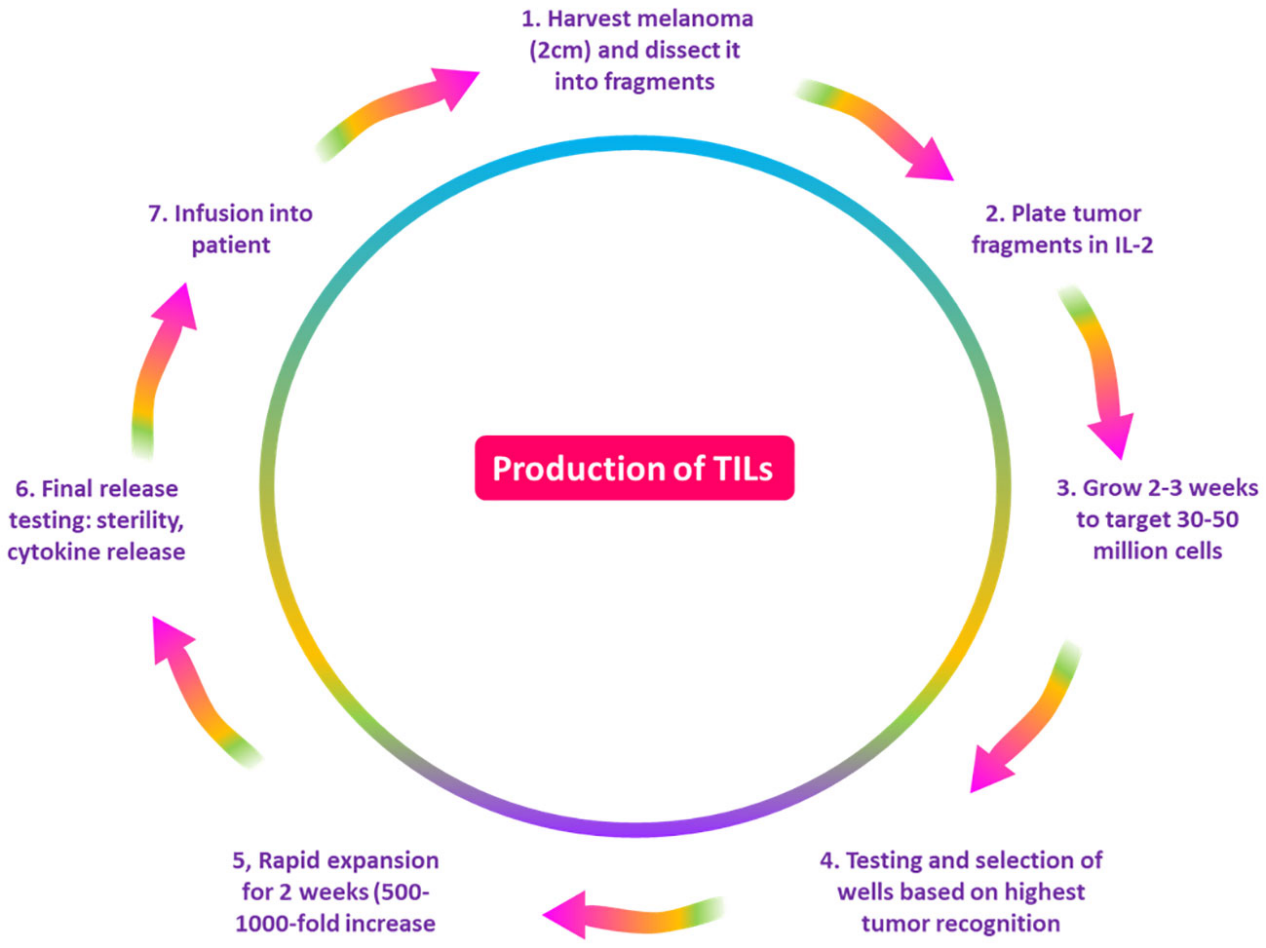
A diagram demonstrating the process of the production of TILs. The NCI/NIH Surgery Branch developed the first techniques for producing lymphocytes that penetrate tumors. First, the patient’s tumor is removed, cut into pieces, and then it is cultured with IL-2 for two to four weeks, or until the number of lymphocytes reaches 30 to 50 million. Following a test against tumor cells, lymphocytes are rapidly expanded for two weeks to reach a target of 50 billion cells, after which they are pumped into the patient. TILs, tumor-infiltrating lymphocytes; NCI, National Cancer Institute; NIH, National Institutes of Health; IL-2, Interleukin 2.

It is believed that immune cells in the TME and cancer play a critical role in regulating the growth and spread of malignant tumors. As a result, TILs have been found in metastases of several cancer types as well as primary tumor tissue and lymph nodes that harbor tumors (38). TILs are a polyclonal population comprising CD3+ T-cells (CD8+ and CD4+ T-cells), FOXP3+ regulatory T-cells, and CD20+ B cells (39). Research has been conducted to look into TILs’ prognostic significance in melanoma (40). TIL treatment is a form of immunotherapy in which a biopsy or surgical procedure is used to separate TILs from the tumor location (**Figure 3**) (41). Interleukin-2 (IL-2) is used to cultivate these cells *in vitro*. By stimulating CD4+ T-cell proliferation and T-helper cell differentiation, IL-2 enhances the antitumor response (42). Additionally, it has been demonstrated that IL-2 can boost natural killer (NK) cells’ cytotoxic potential and CD8+ T-cells’ antitumor attribute (43). The patient receives another dose of the prepared vaccination (41). A TIL- based drug takes 5-7 weeks to create, and it needs specialized machinery and skilled labor (44).

In 1988, Steven Rosenberg was the first to show through a clinical trial that a TIL-based approach might effectively cure melanoma (45). Twenty patients with metastatic melanoma in that research trial were given an infusion of a cultured solution containing TILs and IL-2. Tumor regression was thus seen in 2 out of 5 patients who had previously had therapeutic failure with IL-2. Furthermore, tumor regression was reported in 9 out of 15 people who had not previously received IL-2. Furthermore, regression of tumors was noted in the skin, bones, liver, lungs, subcutaneous adipose tissue, and liver. It was observed that concurrent delivery of IL-2 and previous cyclophosphamide or radiation therapy are major factors in TIL efficacy. The majority of TILs exhibited a CD3+ phenotype, however, patient-specific CD4+ and CD8+ cell counts varied (45). The efficacy of TIL therapy in treating metastatic melanoma patients has been demonstrated by clinical trials, especially in those who are not responding to standard treatments. About 20% of patients saw a full response, indicating the promise of TIL therapy as an individualized immunotherapy. About 50% of patients presented a partial response (37, 46). TIL treatment can extend the lives of people with metastatic melanoma. This is demonstrated by the fact that individuals treated with it had a median overall survival (OS) increase of 6 to 12 months when compared to those receiving standard therapies (15).

## 3 Prognostic value of TILs in melanoma

### 3.1 TIL assessment in hematoxylin and eosin

After Clark and colleagues initially described TIL in melanoma (47), several researchers found a correlation between lymphocyte infiltration and increased patient survival (**Table 3**) (59). Even though studies have revealed that immune cell subsets within human malignancies are extremely diverse, counting TIL using hematoxylin and eosin (HandE) offers crucial information on the TME in some cancer types (60, 61). Based on histology, the immune infiltrate’s location can be classified as intratumoral, stromal, or peritumoral (61, 62). Intratumoral immune cells are found right inside the tumor cells’ malignant “nest” (61). The stromal region is made up of different immune cells, connective tissue, and blood vessels. The invasive front refers to the tumor’s outside edge (62). Thus, the cells in the stroma, neighboring non-involved tissue, and the area surrounding the invasive front can all be referred to as peritumoral (61, 62). There is not yet a single, comprehensive method for determining the total amount of immunological infiltrates in solid tumor tissue stained with HandE. It is possible to evaluate and analyze intratumoral and peritumoral lymphocytes for association with different clinical indicators (61).

**Table 3:** Studies in the literature looking at the relationship between overall survival (OS) and TILs in primary melanomas.

Research study	Patients enrolled	Inferences
van Houdt et al. (48)	237	In 1992, the sentinel lymph node which is the lymph node in close proximity with the primary melanoma site on the direct drainage pathway, was developed and used for intraoperative identification. Consequently, this method accurately determines individuals with early-stage melanoma who have nodal metastases and are probably candidates for radical lymphadenectomy.
Han et al. (49)	1865	TIL grade is an independent predictor of survival and SLN status for melanoma patients; a significant TIL infiltration is linked to a good prognosis.
Vrbić et al. (50)	264	A brisk infiltration was linked to better 8-year survival in contrast to a non-brisk or absent infiltrate, and the presence of TILs was substantially associated with a better prognosis when compared with the absence of TILs.
Berk et al. (51)	327	Thickness in conjunction with biologically based markers like VGP, TILs, and mitotic rate can be utilized to determine the likelihood of SLN positive. This prediction model can be used to choose people who will or won’t have an SLN biopsy if it is validated.
Nikolin et al. (52)	107	This study highlights how crucial the tumor’s anatomical locations are in determining a patient’s prognosis when they have malignant melanoma.
Azimi et al. (53)	285	Comparing thickness, mitotic rate, and TIL presence in a multivariate analysis revealed that only thickness and TIL presence were significant and independent favorable histologic prognostic variables.
Gangi et al. (54)	515	TIL response was a significant predictor of SLN metastases in multivariate analysis, although it was not a significant independent factor predicting DFS or OS.
Francken et al. (55)	3330	In primary melanoma patients, a higher TIL grade is linked to a higher prognosis. Therefore, the TIL grade merits more research to ascertain whether or not it needs to be incorporated into the next AJCC staging modifications.
McMasters et al. (56)	887	In individuals with initial cutaneous melanoma, the presence of ulceration, elevated Breslow thickness, male sex, and lack of TILs are predictive factors for SLN metastases.
Tuthill et al. (57)	1998	Both patient variables (age, anatomic localization) and histological factors (Breslow thickness, ulceration) are significant independent predictors of survival and recurrence. These factors can be used to stratify prognosis in patients with tumor-negative SLN and develop long- term follow-up regimens.
McMasters et al. (58)	1251	The primary predictor of disease-free and OS in a multivariate analysis was SLN metastasis, which is predicted by the absence of TILs.

TILs, Tumor-infiltrating lymphocytes; VGP, Vertical growth phase; SLN, Sentinel lymph node; DFS, Disease-free survival; OS, Overall survival; AJCC, American Joint Committee on Cancer.

It is important to remember, though, that digital full slide images are currently used for the great majority of pathological studies (63, 64). This enables dynamic zooming and panning, high-quality imaging, and the ability to use image analysis software to examine the images (63). Typically, TIL scoring in cutaneous melanoma is limited to spherical inflammatory cells in the intratumoral area, eliminating polymorphonuclear cells (65). Additionally, whereas primary melanoma tumors have been the subject of the majority of TIL investigations, several researchers have also investigated the prognostic significance of TIL examination in metastatic biopsies (66–68).

**Figure 4:**
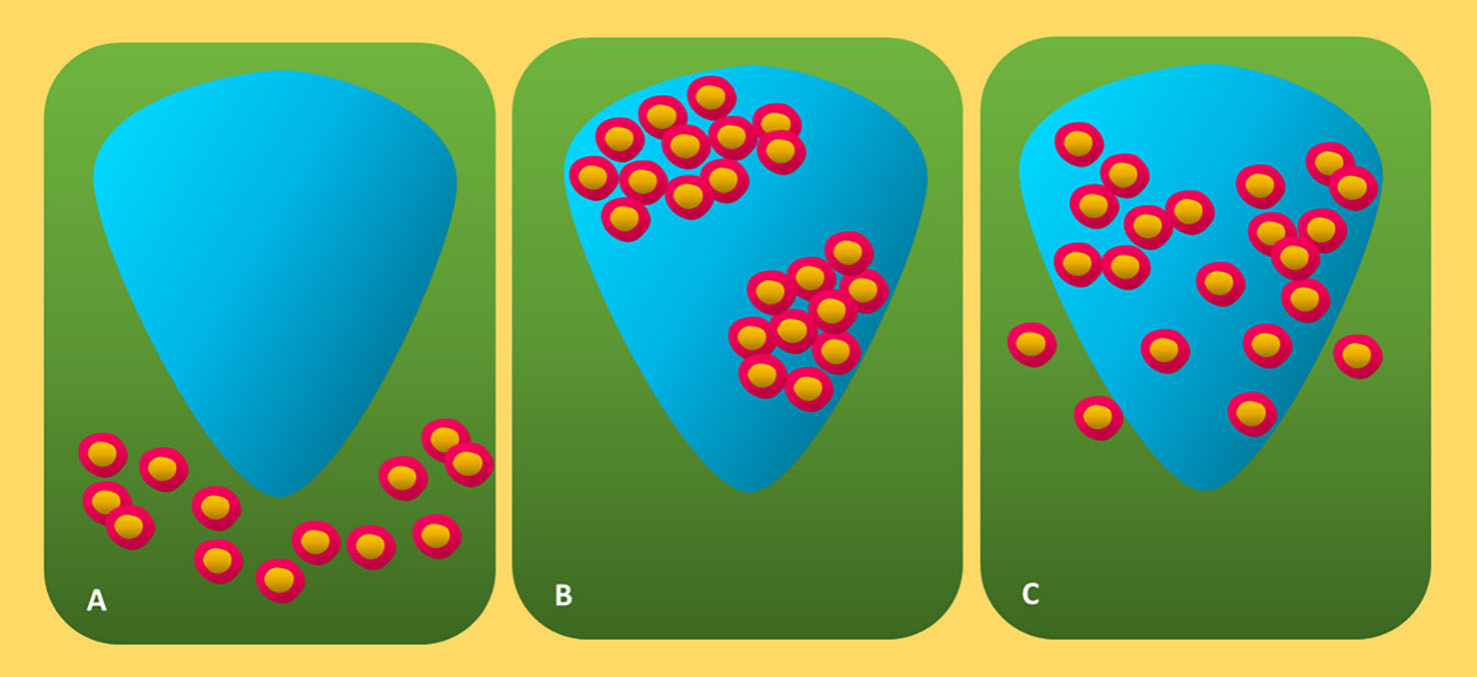
Diagrammatic representation of three forms of canonical TIL invasion in cutaneous melanoma. **(A)** Absent: Neither lymphocyte infiltration nor lymphocyte presence within the tumor. **(B)** Non-brisk: One or more dispersed lymphocyte foci. **(C)** Brisk: Diffuse lymphocyte infiltration near the tumor base or during the tumorigenic vertical development phase. TIL, tumor-infiltrating lymphocytes.

These days, the Melanoma Institute Australia (MIA) approach and the Clark method are the two main techniques for grading TIL in HandE-stained melanoma tissue (40, 53, 61). The Clark score method was first published in 1989 and defines three unique TIL patterns: absence, non-brisk, and brisk (**Figure 4**) (47, 63).

Absent denotes the unavailability of TILs or their inability to invade the tumor (47, 65). Nonbrisk indicates one or more dispersed lymphocyte foci (47, 59). Diffuse lymphocyte infiltration along the tumor’s base or during the tumorigenic vertical growth phase (VGP) is referred to as brisk (47, 60, 69). The brisk TIL patterns scoring was further separated by Clemente and colleagues into two categories: diffuse (infiltrate the invasive region of the tumor entirely) and peripheral (along the tumor base) (60, 70). Lymphocytes in fibrosis-affected areas and perivascular lymphocytes are often excluded from the scoring (60). Due to its great interobserver agreement, reproducibility, and ease of application, the Clark system is still widely used (60, 71). Based on the distribution (diffuse, focal, or multifocal throughout the entire tumor) and density (marked, mild, or moderate) of TIL in the dermis, the MIA grading system is used (53, 60). The definition of the MIA ordinal score (0–3) is as follows: TIL absent in grades 0; mild multifocal or moderate/mild focal infiltrate in grades 1 and 2; marked multifocal or moderate, mild diffuse, or marked focal TIL pattern in grades 3 and 4 (53).

It has been shown by the MIA and Clark scoring systems that higher TIL levels are linked to better prognosis. Clark and colleagues examined the 8-year survival of more than 200 individuals who had primary cutaneous melanoma with various histologic subtypes. Eight-year survival rates for patients with absent TIL were 59, for those with non-brisk TIL patterns it was 75%, and for those with brisk TIL patterns, it was 88% (47). The association between the absence of TIL and a greater number of sentinel lymph node (SLN) metastases is further proof of the biological significance of TIL scoring (72), as defined by Clark and colleagues (47). In melanoma, SLN status continues to be the most significant independent prognostic predictor (73). Using the MIA scoring system, Azimi et al. (53) demonstrated that the TIL grade was independently linked with disease-specific survival (DSS) and inversely associated with SLN-positive in primary cutaneous melanoma (53). There is enough data to show that the presence of TIL is associated with a better prognosis for patients because of the increased host response to the tumor. A meta-analysis of 41 studies on TIL in melanoma revealed that the mere existence of TIL was highly related with enhanced OS. Most of the primary cutaneous melanoma research that was included in this meta-analysis was carried out. The authors provided evidence that brisk TILs were linked to better OS, DSS, prognosis, and recurrence-free survival (RFS) (40). Taken together, these findings show that lymphocytes in the tumor are indicative of the host immune system’s reaction to the malignancy and are, thus, typically linked to favorable clinical outcomes. The International Immuno-Oncology Biomarker Working Group (IOBWG) has suggested a more uniform method for TIL evaluation due to the usefulness of the HandE assessment of TIL (74).

To ascertain this approach’s efficacy for prognosis, melanoma, and other solid tumors must be validated first. Although TIL scoring has shown to be an effective predictive method for melanoma, the immune cells that infiltrate a tumor exhibit a varied range of phenotypes and functions (75). Three main kinds of immune infiltration were determined through histological examination, even though the TME for the majority of malignancies is extremely variable. Tumors with non-T cell-inflamed (cool) and T cell-inflamed (hot) TME can be broadly categorized (76). Elevated IFNγ signaling, PD-L1 expression, a high mutational burden, and a high density of CD8+ TIL are all present in T cell-inflamed malignancies, according to profiling of these subtypes (76, 77). Immunosuppressive myeloid cells or stroma prevent T cells from entering the tumor bulk, resulting in a peripheral buildup of T cells in immune-excluded malignancies (77). On the other hand, cold tumors could be an immune-ignored phenotype with minimal or nonexistent T cell infiltration, significant tumor cell proliferation, no expression of PD-L1, and a low burden of mutations (76, 77). The general categories that are presented here are obtained from tumor tissue histological investigations (77). It is necessary to ascertain the functions of stromal cells, tumor-infiltrating immune cells, and mutational burden in determining the TME phenotype (77, 78). Therefore, one of the most important methods for both pre-clinical and clinical cancer research is the use of IHC for the identification and counting of the primary lymphocyte subsets in the tumor and the assessment of their association to treatment response or patient survival (78, 79).

### 3.2 Prognostic implications of different TIL subtypes

#### 3.2.1 CD8+ TIL

CD8+ T cells play a crucial role in the adaptive immune response against malignancy (80–82). Since activated CD8+ T cells may directly identify and eliminate cancerous and contaminated cells, they are also called cytotoxic T lymphocytes (CTLs) (80, 81). CTLs are the main immune cells that have the ability to regulate tumor growth and mediate responses to cancer immunotherapies. This is accurate despite the fact that certain immune cells, such as macrophages and NK cells, have the ability to cause tumors (81–83). The primary anticancer function of CTL is the production of cytokines such as IFNγ and tumor necrosis factor (TNF), in addition to the exocytosis of cytotoxic granules that include granzymes and perforin (81, 82). CD8+ T cell priming and recruitment in the TME were reported to be dependent on a subgroup of conventional dendritic cells (cDC), known as cDC1 (84). According to the conceptual framework of cancer immunoediting, the tumor can elude the immune onslaught and foster an immunosuppressive TME following a time of early T cell control (80, 85). Conversely, long-term exposure to antigens, as seen in the setting of cancer and chronic viremia, induces T-cell exhaustion. This condition is characterized by a loss of effector capabilities, unique transcriptional patterns, and a persistent expression of inhibitory surface receptors (like PD-1) (86). Consequently, the translation of histological investigations on CD8+ TIL in tumor tissues is complicated by their varied morphologies and functional profiles in the TME (61, 66).

Early analysis indicated that a group of 47 individuals with primary cutaneous melanoma tumors had higher CD8+ TIL levels, which were related to a higher chance of survival. Upon utilizing CD8+ T cell density to stratify the patients, it was observed that the 5-year OS for the low-, moderate-, and high-density cohorts were 25, 44, and 78%, respectively (87). However, in 2011, a larger cohort of more than 180 primary cutaneous melanoma samples revealed no association between patient survival and CD8+ TIL (88). These investigations thus highlight the difficulty in utilizing immunohistochemistry to assess the CD8+ TIL prognostic significance. Numerous studies have looked at the TIL characteristics of metastatic lesions in addition to original tumors. These results show a significant correlation between survival and elevated CD8+ T cell density in Stage III and IV metastatic melanoma tumors (67, 68). As was previously mentioned, CD8+ TIL express a broad range of surface markers and release several functional chemicals (82). Therefore, while investigating the prognostic importance of CD8+ TIL, some research has looked at the utilization of labeling effector molecules or activation markers in addition to identifying CD8. It was demonstrated that TIL positive for the CD8+ T cell effector molecule called Granzyme B (GZMB) was linked to longer O and progression-free survival (PFS) in the Stage II primary melanoma tumors cohort. Additionally, GZMBC TIL were proven to be positive for CD8 by dual IF labeling (48).

Research on the functional condition of T cells in the TME has revealed many biologically significant surface indicators that may help evaluate the prognostic significance of CD8+ TIL in melanoma. Better clinical results in cutaneous melanoma are linked to the presence of the chemokine receptors CCR9 and CXCR3, as well as the c-type lectin NKG2D, on CD8+ TIL. This was corroborated by a flow cytometric analysis of intratumoral CD8+ T cells in metastatic lymph nodes (mLN) (89). The difference between tumor-specific lymphocytes (i.e., those that detect tumor antigens) and bystander TIL is crucial in the TME. In lung and colon cancers, surface-expressed ectonucleotidase CD39 has been shown to be a useful marker for distinguishing tumor-specific CD8+ TIL. This was found by mass cytometry and multiplexed tetramer-based methodology (90). The researchers discovered that T cell immunoreceptors with Ig and ITIM domains (TIGIT), which have been shown to differentiate tumor-specific “exhausted” TIL, and inhibitory receptors like PD-1 are present in bystander TIL (90, 91). The CD8+ TIL diversity in the TME has been shown by sophisticated immunophenotyping technologies. Labeling other markers on CD8+ TIL, such as CD39, TIGIT, or NKG2D, may or may not help predict disease in research and clinical settings, but this is still up for debate. However, when assessing the prognosis of a disease or the potential efficacy of immunotherapy, examination of CD8+ T cells in the TME continues to be one of the most important readouts for intratumoral immune activity (61, 92).

#### 3.2.2 CD4+ TIL

CD4+ T helper (TH) cells are extremely varied and have a phenotype and function similar to CD8+ T cells (93). Moreover, through recently studied pathways, CD4+ T cells are critical for cancer immunity (93, 94). CD4+ T cells have the ability to kill tumor cells directly and cytolytically, as well as create cytokines like IFNγ that strengthen the immune system’s ability to fight cancer (80, 93). Moreover, CD4+ T cells in secondary lymphoid organs can regulate CD8+ CTL and B cell responses. Studies on murine models have demonstrated that CD4+ T cells can boost CD8+ T response efficacy (94). The differentiation of CD4+ T cells into discrete T helper lineages identified by unique transcription factors and cytokine production has also been demonstrated (93, 95). The lineages T follicular helper (TFH), TH1, TH2, TH17, and CD4+FOXP3+ Treg are the most studied of these. It is widely acknowledged that Th1 cells exploit their high IFNγ production to support efficient immune responses against tumors. Consequently, it recruits NK cells and M1 macrophages that have been classically activated in addition to modulating CD8+ TLR activity. Nevertheless, the complex roles that each subgroup plays in various tumor types are still not well understood and need more research (93). Alternatively, Tregs are thought to promote tumorigenesis because of their ability to reduce immunological responses and block effector T cells (96).

As previously indicated, the majority of research on TIL’s prognostic significance in melanoma has used TIL pattern scoring in HandE tissues. Thus, a relatively handful of investigations have looked at TIL subgroups *in situ* using IHC (61, 66, 79). Using Stage II primary cutaneous melanomas, Van Houdt et al. (48) demonstrated that CD8+ and CD4+ TIL were only associated with improved PFS and not OS, but GZMBC TIL was associated with both prolonged OS and PFS (48). Nevertheless, there are not any solid studies showing the prognostic value of CD4+ TIL evaluation utilizing histopathology in the setting of metastatic melanoma (61, 66). Jacquelot et al. (89) discovered an inverse relationship between the frequencies of CD8+ T cells and the fraction of naïve CD45RA+CD4+ T cells using multi-parameter flow cytometric profiling. This was found in the mLN of patients with stage III cutaneous melanoma. Furthermore, it was observed that patients whose tumors had notably higher percentages of naive CD45RA+CD4+ T cells had significantly shorter PFS. Finally, it was found that CD4+ T cells in metastatic tumors additionally expressed surface markers, PD-1 and CD69. Nevertheless, it is still unclear if evaluating these markers *in situ* IF or by immunohistochemistry has any predictive value for melanoma (89).

Very little research has used IHC or IF to examine the predictive significance of CD4+ TIL enumeration in melanoma (61, 66). Higher concentrations of CD8+ and CD3+ TIL were successfully linked with OS, but not CD4+ TIL. This was demonstrated by studies that used TMAs created from several anatomic regions of metastatic melanoma samples and IHC to identify the primary TIL subgroups (67). However, in a study that looked only at melanoma metastases inside the SLN and visually counted intratumoral cells, greater CD4+ TIL counts were substantially linked with improved OS and RFS (68). The investigation of distinct TIL subsets within the metastatic SLN can yield valuable insights into the molecular and prognostic aspects of the functions these cells perform in cancer immunity. This is because primary cutaneous melanoma is commonly staged via SLN biopsy (97). The aforementioned data, however, make it more difficult to understand how CD4+ TIL functions in melanoma. Firstly, standardized comparisons across numerous findings are not possible due to the limited number of research looking at melanoma TIL subsets and the variation in methods being utilized to determine and count labeled cells (61, 66). Second, identifying merely the CD4 surface antigen is insufficient to describe CD4+ T helper cell diversity. Although TH1 CD4+ TIL is thought to boost cancer immunity, the functions of TH17 and TH2 are extensively complex. Furthermore, it is unclear how they play a part in the formation and spread of tumors (93, 98). The understanding of how T helper subsets impact tumor formation has primarily come from *in vivo* mouse models, whereby B16 melanomas have been demonstrated to be eradicated by CD4+ TH1 and TH2 cells (98). However, because T-helper subpopulations are generally defined by the differential expression of important cytokines rather than the expression of distinctive surface markers, investigating them in the context of immunopathology is difficult (93). This has led to several investigations using gene expression profiling to evaluate the presence of TH2 or TH1 hallmark genes in human melanoma samples. According to a report, primary melanoma tumors that spontaneously regress (a clinically confirmed event suggesting host anti-tumor immune response activation) express significantly more TH1-linked genes, such as IL-2 and TNFβ, than tumors that do not. Regressing primary tumors also showed increased gene expression of IFNγ, which is the main TH1 effector cytokine. However, these levels did not become statistically significant (99).

In another investigation, VEGFA production was observed to be greater in CD4+ T cells obtained from the SLN of 13 patients with cutaneous melanoma who had TH2-skewed gene profiles. In this study, both positive and negative SLN were included (100). While the exact mechanism of T cell differentiation into distinct effector subpopulations remains unknown, it is known that specific transcription factors regulate the differentiation of CD4+ T cells into distinct T helper lineages. These include TH1 (T-bet), TH2 (GATA-3), Treg (FOXP3), and TH17 (RORgt), As a result, T-bet+ cell IHC has occasionally been used as a readout for TH1 cells in tumor tissue. Increased levels of T-bet+ cells have been correlated with better patient outcomes in both ovarian and colorectal primary cancers (101, 102). Nevertheless, no comparable research on melanoma has been published in the literature (40, 61, 66). Moreover, studies showing Treg to be T-bet expressing raise doubts regarding the biological validity of using T-bet in dual or single-marker IHC to identify TH1 cells. Levine et al. (103) demonstrated in a seminal study that Treg cells express T-bet consistently in murine models, and that the reduction of T-bet+ Treg in mice led to strong TH1 autoimmune reactions (103). As a result, to evaluate CD4+ TIL in cancer tissues and determine the prognostic and biological significance of each subset, it will be necessary to assess several distinct phenotypic markers in addition to CD4+. This is because earlier studies have cast doubt on previously accepted assertions regarding T helper cell differentiation. This is particularly true given the variety of Treg populations found in cancers and the varying findings from research using FOXP3+ to look at Treg that have penetrated tumors (79, 104).

#### 3.2.3 Natural killer cells

The innate immune system’s effector cells, known as NK cells, are crucial for eliminating tumor and virus-infected cells (105). Additionally, through their interactions with DCs, they take role in the control of adaptive immune responses (106). A balance of signaling pathways initiated by activating (NKG2D, DNAM-1, NKp30, NKp44, NKp46, etc.) and inhibitory receptors (CD94/NKG2A, members of the killer cell immunoglobulin-like receptor family, etc.) receptors determines the activation state of NK cells. Since they are typically scarce in solid tumors, NK cells are thought to primarily contribute to systemic antitumor defense by preventing the growth of hematogenous metastases. Additionally, reduced functional activity and low activating receptor expression are common characteristics of tumor-infiltrating NK cells (107, 108). The impact of immune-suppressive substances generated by tumor cells or nearby stromal cells, which have been reported in a number of tumor forms, including melanoma, may be the source of this (109, 110). Conversely, in certain tumor forms, a higher number of NK cells was linked to a better prognosis (107, 111). However, it should be mentioned that the majority of research used antibodies against the CD56 or CD57 markers, which are expressed by cell types other than NK cells. In contrast, the use of the NKp46 marker, which is thought to be more NK-specific, has only begun to gain traction afterwards (108, 112). A smaller proportion of CD56^bright^ NK cells in melanoma patients is associated with a shorter overall and PFS, even though there is no overall difference in the number of circulating NK cells between healthy donors and melanoma patients (113). In stage IV melanoma, the same relationship was seen for NKp46 on circulating NK cells, where more expression was linked to prolonged survival (114). Nevertheless, according to de Coaña et al. (115), there was no relationship between NKp46 expression and OS; instead, low CD69 expression might be a predictor of improved OS (115). Using a different approach, analyzing RNA-seq data from bulk tumor samples, Cursons and colleagues used a NK cell signature to predict NK cell infiltration into melanoma and showed that this correlated with a better survival (116).

#### 3.2.4 B lymphocytes

B lymphocytes are often seen in relatively small quantities in solid tumors, with a few exceptions. It is unclear how antibody-dependent immunity contributes to the immune response against tumors, and it is still up for debate how the biological activity of tumors is influenced by systemic B-cell response or *in situ* B-cell accumulation. B lymphocytes have been demonstrated to promote tumor growth or progression in several experimental tumor models (117, 118). However, other research produced the opposite findings (119). According to Nelson (120), the variation in B lymphocytes’ systemic pro- or antitumor effects could be linked to the variety of their functional activities. In addition to secreting various immune suppressive substances and supporting tumor growth through antibodies or immune complexes, B lymphocytes may also serve as efficient antigen-presenting cells that stimulate the anticancer T-cell response (120). T cells make up the majority of melanoma infiltrates, just like in other tumor types, with B-cell frequencies estimated to be between 15% and 20% of all infiltrating lymphocytes (121, 122). The vast majority of B lymphocytes expressing the CD20 marker in studies on 106 primary melanomas were found to be peritumoral, primarily distributed in the stroma surrounding tumor deposits. Additionally, B-cells arranged in dense, follicle-like aggregates were seen in roughly 25% of the samples (123, 124). According to different cancer types and melanoma metastases, these B-cell clusters formed ectopic lymphoid structures with T lymphocytes (125–128). Some of the follicle-like structures showed a network of CD21+ follicular DCs, and in most cases, MECA-79+ HEV-like venules were also seen adjacent to these (124). According to Ladányi et al. (123), there was a correlation between the density of activated (CD25+ or OX40+) T cells and B lymphocytes in the melanoma samples. Additionally, peritumoral densities of B-cells and activated T lymphocytes showed a very bad prognosis in the case of low amounts of both cell types, and intensive B-cell infiltration offered a considerable survival advantage (123). Although there was a trend toward a larger incidence of B-cell aggregates in thicker tumors, their appearance did not correlate with the disease’s outcome (124). The quantity of invading B lymphocytes with a predictive impact was not discovered in a previous investigation with less cases (121).

## 4 Clinical implications and applications

### 4.1 TILs as an immunotherapy biomarker

For melanoma, breast cancer, endometrial cancer, colorectal cancer, and non-small cell lung cancer (NSCLC), higher baseline TIL density is linked to better outcomes (ORR in metastatic and pathological complete responses (pCR) in early illness). These solid tumors were treated with ICIs (129–143). Moreover, studies assessing histological samples obtained during treatment demonstrated that changes in TIL density throughout treatment were linked to better results, even in cases where no correlation with baseline TILs was seen (132, 144). The reaction to ICI is also influenced by spatial dispersion in addition to TIL density and change in the TME. Due to its demonstrated ability to offer extra information on early alterations following ICI administration in and around the tumor, the ratio between the invasive margin (IM) and the tumor center (CT) may be of particular relevance. It may also serve as an early predictive biomarker for the effects of treatment (133, 134, 139, 145–147). Findings like these show that the tumor-host environment is a dynamic system that constantly adapts to changes. Furthermore, in patients undergoing ICI treatment, the dynamics in TILs, particularly during and after treatment, may be predictive and prognostic (38).

The impact of acidity and hypoxia on TIL effector proliferation and function further demonstrates the critical role of TME in TIL dynamics. According to certain theories, an acidic environment inhibits IL-2’s stimulatory function, stopping lymphocyte proliferation (148–150). Moreover, CD8+ T cells’ cytolytic activity and cytokine release are hampered by acidosis in the TME (149, 151). Hypoxia, which is frequently brought on by disorganized and inadequate tumor microcirculation (152), impairs the ability of CD8+ and CD4+ T lymphocytes to perform effector tasks and promotes the growth and migration of immune-suppressive cells. Moreover, Zandberg and colleagues reported that the impact of anti-PD-1 therapy is weakened by elevated hypoxia inn HNSCC (153). TIL subsets (e.g., CD8+, Tregs, CD4+, and T-memory), exhaustion markers (e.g., LAG-3, PD-1, and TIM-3), and activation markers (e.g., granzyme B) as well as their link with clinical outcome post ICI treatment were investigated. This is in addition to the overall predictive and productive significance of TILs (140, 141). Although not conclusive as a prognostic biomarker, these studies demonstrate the relationship between TILs and TME after ICI treatment. Additionally, the predictive significance of a TIL varies according to the ICI employed (141). While TILs in tumors may predict ICI treatment, many studies lack an ICI-free arm for reference, making it difficult to draw definitive conclusions. High TIL infiltration and exhaustion/activation indicators are not always associated with a positive or negative response. In ovarian cancer, immune treatment trials have shown minimal response to ICI, even in tumors with significant TIL concentrations (154, 155).

## 5 Current challenges and limitations of TIL-based therapies

TIL-based therapy is not yet the preferred treatment for metastatic melanoma patients, since it has been recently approved, and only a few patients have been treated. ICI continues to be the main therapeutic option for metastatic melanoma patients as detailed in **Table 2**. Treatment-associated adverse events, treatment resistance, and TIL synthesis requirements are some of these issues. First, only well-funded medical facilities with the capacity to accommodate the required technologies to manage the TIL workflow and any treatment-emergent adverse events (TEAs) that can arise during patient hospitalization are largely able to produce TILs due to their labor-intensive and complex nature. Expanding the production of TIL products centrally is essential because it may enable the use of TIL-based ACT widely and affordably. One example of this is Iovance Biotherapeutics, which developed a central manufacturing facility to produce Lifileucel, an autologous TIL product that is cryopreserved (37, 156). The FDA’s recent approval of lifileucel (Amtagvi) for advanced melanoma seems encouraging (157).

Second, to get reinfusion, the patient must also follow a pre-conditioning program, the majority of treatment-related toxicities being recorded as side effects. These toxicities fall into the category of uncommon autoimmune-related toxicities, which are usually caused by tumor-linked antigens on healthy cells expressing non-specifically. The reintroduction of lymphocytes or cytokine-related toxicities, which are caused by the high doses of IL-2 that are frequently administered with TIL therapy to enhance the lymphocytes’ anticancer activity, can target these antigens (44, 158). It has been shown that this preparative lymphodepleting treatment, which consists of fludarabine and cyclophosphamide, increases the efficiency of TIL-based ACT. Nevertheless, cellular processes behind this effect are still unclear, and there is a significant trade-off in terms of severe toxicities (159).

Lastly, resistance to TIL use is possible, as it is in many situations. There is a high prevalence of both innate and acquired resistance. Innate resistance is defined as the patient’s observed lack of reaction after the first therapeutic administration, while acquired resistance is the developed resistance that appears following a patient’s prior positive response (156). There are three primary distinctions in the mechanisms resulting in different resistance forms: (1) TME-driven T-cell depletion and/or malfunction; (2) immune-suppressive cells interfering with TME-driven T-cell recognition; and (3) limitations on T-cell migration to the tumor (156, 159). Even while our understanding of the mechanics driving these resistance occurrences is growing, more has to be done to improve TIL curation. Current research has shown promise in combining TIL therapy with other treatments. One such study is the randomized prospective phase II trial (NCT02621021), which is being carried out to find out if patients with metastatic melanoma may experience higher response rates when pembrolizumab is added to TIL/IL-2 therapy (160).

## 6 Future directions and research priorities

### 6.1 Advanced techniques for TIL characterization

An analysis of the TIL profiling literature reveals that determining TIL characteristics and density provides a valuable mechanistic understanding of tumor immunology (60, 61). Furthermore, it was reported that TIL profiling provides predictive information about a successful response to ICI in addition to prognostic value for patient outcomes (61, 161, 162). Yet even when it comes to well-researched tumor forms like melanoma, the literature also shows that there is disagreement on how to identify, count, and score TIL subtypes (61, 66). Furthermore, highly multiplexed techniques are needed to find novel spatial correlations and phenotypic features. Therefore, two important facets of advanced characterization techniques in cancer and their application to cutaneous melanoma are discussed below. “Immunoscore” is another scoring system for immune cells that infiltrate tumors is initially explored. This algorithm has demonstrated exceptional predictive potential and reliability in cases of colorectal cancer (163, 164). Secondly, *in situ*, immunophenotyping techniques that show a lot of promise for revealing the TME’s complexity, especially its immunological background are explored.

#### 6.1.1 Immunoscore

Currently, the IHC-based identification of many TIL-specific markers is one of the main methods for identifying the lymphocyte infiltrates in the TME (including FOXP3, CD3, and CD8) on successive tumor tissue slices (61, 79). Therefore, measuring lymphocyte densities as ratios like CD4/CD8 or CD8/FOXP3 was studied and has demonstrated prognostic value in other types of tumors. However, it still needs to be validated and thoroughly examined (165, 166). Nevertheless, a brand-new intratumoral lymphocyte scoring system called the “Immunoscore” has emerged as an effective predictive tool in colorectal cancer. According to digitally quantified densities of IHC-labeled CD8+ and CD3+ T cells in the CT and at the IM, immunoscore is a scale that ranges from I0-I4 (164, 167). Using specialist image analysis software (Immunoscore^®^ Analyzer, HalioDx, France), an operator first recognizes specific locations (tumor, necrosis, healthy tissue, etc.), and then confirms the CD8 and CD3 stains. The software automatically determines an IM that extends 360 mm into the healthy tissue and 360 mm into the malignancy. Immunoscore outperformed the TNM staging system used by the AJCC/IUCC (American Joint Committee on Cancer, International Union for Cancer Control) in stage I, II, and III colorectal cancer. This relates to DFS, OS, and DSS prognostic variables. The primary tumor (T), involvement of the LN (N), and metastases (M) are examined by the TNM staging system (167). Tissue biopsies from more than 2,500 patients with colorectal cancer were examined by Immunoscore in a worldwide multi-center study spanning more than 13 nations. The results showed that the test had excellent repeatability and consistency among various sites. In contrast to patients with a lower Immunoscore, those with an elevated Immunoscore had a considerably lower chance of experiencing a disease recurrence after five years (163).

Attempts were made to apply Immunoscore to other tumor types. These include melanoma, non-small cell lung cancer (NSCLC), and breast cancer, due to its effectiveness in colon cancer. As a result, efforts to clinically validate Immunoscore as a prognostic and predictive marker (for ICI) in various other tumor types are still ongoing. TIL markers including FOXP3 and CD20 have also been added to the Immunoscore evaluation in melanoma (168). Overall, the potential for prognosticating clinical outcomes or ICI predictive efficacy of incorporating new immune markers into a more thorough Immunoscore may be greatly increased, given that Immunoscore has not yet been well investigated for melanoma and several other tumor types (39).

#### 6.1.2 Advanced TIL characterization

When novel imaging and molecular tools become available, the capacity to examine many signals simultaneously offers enormous promise for the identification of actionable and clinically significant immune markers in cancer. Single-molecule fluorescent *in situ* hybridization (FISH) has made it possible to evaluate numerous RNA molecules at a single-cell resolution, according to studies. A recent report also showed how to multiplex the profiling of up to 40 proteins at once using an iterative immunofluorescence (4i) based method (169, 170). These technologies are still in the early stages of research; therefore, it is unknown how well they will work for *in situ* tumor tissue immunophenotyping. Many new image analysis software and detection techniques, such as nucleotide tagging, cyclic immunofluorescence, or metal ion tagging, have been recently developed. This is for the simultaneous detection of numerous protein markers (171). The GeoMx™ digital spatial profiling (DSP) method, which uses antibody probes or RNA attached to photocleavable oligonucleotides, was also recently disclosed by NanoString Technologies. To see tumor (like PanCK) and immunological (like CD45) areas in fluorescent markers-labeled tissue, UV light is channeled toward specific areas of interest using digital micromirror devices. Nevertheless, although this method permits the simultaneous and spatially resolved detection of several protein markers or RNA in FFPE tissue, it is not capable of multiplex viewing of multiple markers on individual cells (172).

## 7 Conclusion

The various functions of TILs in the dynamics of malignant melanoma progression and their importance for the prognosis of melanoma have been discussed in this review. The genetic alterations and signaling pathways linked to the development and metastasis of melanoma were also discussed. Additionally, the effect of melanoma immunotherapy on TIL biology has been examined. Prognostic implications of different TIL subtypes including CD8+, CD4+, NK cells, and B lymphocytes were also explored. Finally, we highlighted the present obstacles and limitations of TIL-based therapeutics, as well as improved approaches for TIL characterization. Overall, a growing body of research demonstrates the significance of specific TIL indicators for assessing response to melanoma immunotherapy and supports their prognostic significance in melanoma. TILs will become more and more useful biomarkers in upcoming studies by incorporating improved techniques and immunobiological understanding, which will enhance melanoma prognosis and clinical management. The FDA’s recent approval of Lifileucel is a step forward in the treatment of melanoma and improved patient outcomes. Future research is therefore trying to expand TIL treatment to include common epithelial malignancies, which account for more than 80% of all cancer fatalities and for which checkpoint inhibition has had little effect. Because neoantigen recognition is less prevalent in TIL than in melanoma TIL, this has necessitated screening and selection of TIL. Consequently, a number of early achievements have demonstrated the potential and promise of TIL for these illnesses (36).
